# The First Symposium on COPD and Therapeutic Approaches 2016, Tehran, Iran

**Published:** 2017

**Authors:** Esmaeil Mortaz, Hamidreza Jamaati

**Affiliations:** 1 Chronic Respiratory Diseases Research Center, National Research Institute of Tuberculosis and Lung Diseases (NRITLD), Shahid Beheshti University of Medical Sciences, Tehran, Iran; 2 Tobacco Prevention and Control Research Center, National Research Institute of Tuberculosis and Lung Diseases (NRITLD), Shahid Beheshti University of Medical Sciences, Tehran, Iran

Dear Colleagues,

The first ever Meeting in Iran dedicated solely to COPD was held in Tehran, Iran from 26 to 28 Nov 2016. The conference was focused on the unique and challenging interface between chronic obstructive pulmonary diseases and beyond. This 1st Meeting corresponded with World Chronic Obstructive Pulmonary Disease Day which is a global effort to boost people’s understanding of COPD and advocate for better care for patients. It takes place annually on the second or third Wednesday of November.

In recent times, the clinical and respiratory research aspects of COPD and its various systemic manifestations and comorbidities have evolved separately. Today, with the ever growing burden of diseases in modern society, we are once again seeing a convergence of these disease components in relation to personalised treatment. The re-discovered common ground between respiratory and internal medicine aspects of COPD and its complications are gaining interest in relation to novel therapeutic approaches for these patients.

Due to the importance of this concept, Tanaffos, the Journal of the National Research Institute for Tuberculosis and Lung disease (NRITLD), has decided to publish the Symposia outputs as a supplementary issue.

In this supplementary issue, we have put together a truly multi-disciplinary overview that addresses the cutting edge research issues in COPD. Internationally renowned clinicians and scientists, who participated at the symposium, have contributed a series of state-of-the-art peer reviewed articles. These cover all aspects of COPD research from the role of small airways in COPD, using imaging to define neutrophil activation, identification of COPD subphenotypes including ACOS, eosinophilic COPD and mitochondrial dysfunction and how recognizing these may lead to a more personalised approach to treating these patients. Other areas of major interest included the importance of pulmonary rehabilitation, the potential roles of non-invasive ventilation and high flow nasal therapy in COPD and how selecting the correct device for each patient is critical for the success of bronchoscopic lung volume reduction surgery.

Future challenges such as the effect of climate change and air pollution of COPD incidence and severity, imaging of COPD airways and phenotyping COPD treatable traits at the molecular/omic level were also highlighted. Finally, there are reports on progress in biomarkers for the stratification of COPD and outcomes assessment, the relationship between COPD and cardiovascular disease and novel therapeutic approaches including aspects of dietary control and molecular characterisation of food ingredients that may contribute to disease.

We kindly acknowledge all the participants for their effort and believe that COPD remains a pivotal area with a critical need for improved mechanistic understanding to allow improvements in disease management.

